# Human adipose tissue protein analyses using capillary western blot technology

**DOI:** 10.1038/nutd.2017.35

**Published:** 2017-10-02

**Authors:** J Lu, C C Allred, M D Jensen

**Affiliations:** 1 Department of Endocrinology, Changhai Hospital, Second Military Medical University, Shanghai, China; 2 Division of Endocrinology, Diabetes and Metabolism, Endocrine Research Unit, Mayo Clinic College of Medicine, Rochester, MN, USA

## Abstract

**Background/Objectives:**

A capillary western blot (Wes) technology has recently been validated for analyses of cell culture lysate proteins, but whether it is reliable for human tissue proteins is unknown.

**Subjects:**

We compared traditional western blotting to the Wes capillary Western method to quantitate the relative amount of human adipose tissue CD36, the ratio of phosphorylated Erk1/2 (pErk1/2) to total Erk1/2 during insulin clamp or after niacin treatment and the fold increase in pAkt^S473^ (Akt phosphorylation on Ser473) in response to feeding.

**Results:**

The results from these two methods were highly correlated (*r*=0.932 for CD36, *r*=0.905 for pErk1/2:Erk1/2, *r*=0.923 for the change in pAkt/Akt, *P*<0.001). On Wes we observed the distinct peaks around the expected molecular weights for these proteins with decreasing peak areas with serial dilutions of loading protein amount. Wes and traditional western blot both had linear dynamic ranges for CD36, Erk1/2 and Akt. Due to differences in signal responsiveness for pAkt/Akt, we employed a calibrator sample and log transformation of data to allow proper comparisons. The Wes approach required less sample than the traditional western blot and less technician/assay time, while achieving high sensitivity and good reproducibility.

**Conclusions:**

Capillary Western technology (Wes) provides a satisfactory alternative for analyses of human adipose tissue proteins.

## Introduction

The traditional western blot is one of the most widely used analytical techniques to quantitate-specific proteins in tissues and is considered the ‘gold standard’. Recently, a novel capillary electrophoresis-based western blot technology has been developed by ProteinSimple Inc.^1^ This capillary western blot assay incorporates sample separation performed in glass capillaries and immobilization of the proteins directly onto the capillary walls, followed by immuno-probing and chemiluminescent detection. This method has been validated for analyses of cell lysate proteins,^2^ but whether it can be applied to adipose tissue extracts is unknown. Because the amount of published work on human adipose tissue proteins is considerably less than for cell or animal model systems, and because the lipid components of adipose tissue offer some challenges that cell models do not, we wished to test whether the results from this new approach are as robust as those for traditional western blotting. For example, it may be important to understand whether differences in dynamic ranges and/or responses due to the much smaller amounts of sample with the capillary western blot assay require different analytical considerations. If the two approaches give similar results we and other investigators can be confident that data generated from Wes are comparable to standard methods.

We elected to compare these two approaches to measure an adipose tissue protein amount, to measure the ratio of a phosphorylated protein to total protein, and to measure the changes in the phosphorylation state of an adipose tissue protein in response to an *in vivo* stimulus. Our rational was that if Wes and western blotting were in good agreement for these three types of measurements we could have confidence in the technology for future applications.

The first protein we assessed was CD36, a transmembrane protein present in adipose tissue that facilitates cellular fatty acid uptake. The total amount of CD36 protein relates to *in vivo* adipose tissue fatty acid storage.^3^ We also measured the ratio of phosphorylated Erk1/2 to total Erk1/2. Erk is an intracellular signaling pathway included in the mitogen activated protein kinases (MAPKs) family. In adipose tissue, phosphorylated Erk1/2 is necessary to initiate the preadipocyte into the differentiation process.^4^ Erk1 and Erk2 can be activated by insulin and by potentially by niacin acting via the beta-arrestin 1 pathway.^5^ Finally, increases in the phosphorylation status of Akt in response to insulin are thought to regulate glucose uptake and inhibition of lipolysis in adipocytes.^6^ Because of our interest in adipose tissue insulin responses, we tested the ability of Wes to measure the fold increase of pAkt^S473^ (Akt phosphorylation on Ser473) in response to feeding compared to traditional western blotting. Our results indicate the two methods are in excellent agreement provided some aspects of sample loading and data handling are addressed.

## Materials and methods

### Subjects

The studies that were conducted to provide the samples were approved by the Mayo Clinic Institutional Review Board. Informed, written consent was obtained from all volunteers, who were healthy and receiving no medications.

For the examination of adipose tissue CD36, 7 volunteers were admitted to the Mayo Clinic Clinical Research Unit (CRU) the evening prior to the biopsies on two separate occasions. On one study day they underwent a 2 h euglycemic, hyperinsulinemic clamp with an insulin dose of 1.0 mU kg^−1^ min^−1^ and the other study day included a 2 h infusion of somatostatin (200 μg h^−1^) and epinephrine (13 ng kg^−1^ min^−1^). At the end of the infusions on both days we performed abdominal and femoral adipose tissue biopsies.

The samples used to examine Erk1/2 were collected from six volunteers who had undergone a euglycemic, hyperinsulinemic (0.5 μU kg^−1^ min^−1^) clamp and six volunteers who had received two doses of extended-release niacin (1 gram at 0700 hours and another at 0800 hours). Abdominal subcutaneous fat biopsies were collected at 0830 hours^7^ for both groups of volunteers.

For the examination of Akt, eight women (six non-obese) and four men (three non-obese) were admitted to CRU the evening prior to the study. At 0620 hours the participants began consuming small portions of a liquid meal at 20 min intervals in order to increase plasma insulin concentrations as previously described.^8^ Abdominal subcutaneous fat biopsies were collected at 0830 hours (postprandial state). The participants remained in the CRU until the morning when they had repeat adipose tissue biopsies in the overnight fasted state.^9^

We also made a calibrator sample from a pooled whole-tissue extract of adipose tissue from surgical waste. The calibrator sample was used to test the protein detection, assay performance and dynamic range by both western blot and Wes.

### Reagents

Wes-Rabbit (12-230 kDa) Master Kit (# PS-MK04, # PS-MK01) including anti-rabbit secondary antibody, antibody diluent, molecular weight ladder, streptavidin-HRP, dithiothreitol (DTT), fluorescent master mix, luminol-S, peroxide, sample buffer and wash buffer were purchased from ProteinSimple (San Jose, CA, USA). This kit also provides capillary cartridge and pre-filled microplates. Antibodies used were specific for Erk1/2 (#9102), pErk1/2 (#9101), total Akt (#4691), pAkt^S473^ (#4060) (Cell Signaling, Danvers, MA, USA) and CD36 (#sc-9154, Santa Cruz, Santa Cruz, CA, USA). Kaleidoscope pre-stained standards (#161-0324, Bio-Rad Laboratories Inc., Des Plaines, IL, USA) were used as molecular weight marker in western blot.

### Adipose tissue extracts prepared for assays

Adipose tissue from needle biopsies was washed with saline, frozen in liquid nitrogen, and stored at −80 °C. For these assays, tissue was weighed and placed in a tube containing Omni ceramic homogenization beads (Omni International, Kennesaw, GA, USA). Tissue was homogenized in SHBP (20 mm Tris-HCl, 1 mm EDTA, 255 mm sucrose, PH 7.4, and anti-protease cocktail tablet from Roche), 1-4 μl mg^−1^ tissue at 4 °C using an Omni Bead Ruptor (speed=2.10, cycles=2, time=15 s, and delay=10 s). If the extract was prepared for testing pErk1/2 or pAkt, Halt phosphatase inhibitor cocktail (Thermo scientific) was added to the homogenization buffer. The homogenate was centrifuged at 1000 *g* for 10 min at 4 °C and the subnatant (whole-tissue extract) below the lipid cake was aspirated into a new tube. Total protein concentrations of the whole-tissue extracts were measured using Pierce BCA Protein Quantitation kit (Thermo scientific) and the samples were stored at −80 °C until assayed.

### Western blot analyses

Dilutions of whole-tissue extracts with NuPAGE 4 × LDS sample buffer were loaded on 10% NuPAGE Bis-Tris gels (Life Technologies, Camarillo, CA, USA). Proteins were separated by gel electrophoreses running on an Invitrogen Midi Gel system and transferred to polyvinylidene difluoride (PVDF) membranes on an Invitrogen semi-dry apparatus at 20 V for 1 h. The membranes were blocked with 5% nonfat dry milk in TBS (10 mm Tris, 150 mm NaCl, pH 7.4) for 1 h at room temperature. Primary antibodies (1:500 dilution for CD36, 1:1000 dilution for Erk1/2, pErk1/2, total Akt, and 1:2000 dilution for pAKT ^S473^) were incubated overnight at 4° C in TBST (TBS with 0.1% Tween 20) with 5% BSA. After four 5 min washes in TBST, the secondary antibody (Odyssey IRDye 800CW goat anti-rabbit-HRP, Li-Cor Biosciences, Lincoln, NE, USA) was incubated in a 1:10000-1:15000 dilution in TBST with 5% BSA for 1 h at room temperature followed by four 5 min washes in TBST. Detection of immuno-reactive bands was performed by image scan using a LI-COR Odyssey Imaging System (LI-COR Biosciences).

### Capillary western blot (Wes) analyses

Capillary Western analyses were performed using the ProteinSimple Wes System. Samples (whole-tissue extracts) were diluted with 0.1 × Sample Buffer. Then 4 parts of diluted sample were combined with 1 part 5 × Fluorescent Master Mix (containing 5 × sample buffer, 5 × fluorescent standard, and 200 mm DTT) and heated at 95 °C for 5 min. The Fluorescent Master Mix contains three fluorescent proteins that act as a ‘ruler’ to normalize the distance for each capillary because the molecular weight ladder is only on the first capillary and each capillary is independent. After this denaturation step, the prepared samples, blocking reagent, primary antibodies (1:50 dilution for Erk1/2, pAkt^S473^, 1:100 dilution for total Akt, and 1:150 dilution for CD36, pErk1/2, the same antibodies as western blot), HRP-conjugated secondary antibodies and chemiluminescent substrate were dispensed into designated wells in an assay plate. A biotinylated ladder provided molecular weight standards for each assay.

After plate loading, the separation electrophoresis and immunodetection steps take place in the fully automated capillary system.

### Data and statistical analysis

Intensities of bands in western blot and Areas under peaks in Wes were detected to present for the relative amount of special proteins. A linear regression fit was used to determine the linear dynamic ranges of special proteins. Pearson correlation analysis was used to estimate the correlation of results from the western blot and Wes assay. Using the fold results in rank ordinal numbers, Spearman rank correlation analysis was also used to measure the correlation of results from these two assays. A *P*-value of Correlation coefficient <0.05 was considered significant. Statistical tests were performed with SPSS version 19.0 for Windows (SPSS Inc., Chicago, IL, USA).

## Results

### Operational principle of Wes

Approximately 40 nl loading sample is injected into the capillary. Proteins are separated in capillaries as electrophoresis causes migration through a stacking and separation matrix.^10^ The separated proteins are immobilized to the capillary wall via UV light cross-linking, following which the capillaries are washed with buffer to remove the gel matrix and are blocked with antibody diluent. The protein of interest is immuno-probed using a specific primary antibody (recommended dilution of 1:50-1:150), followed by an HRP-conjugated secondary antibody and the luminol-S/peroxide substrate. Data analysis is performed using the Compass Software (ProteinSimple). Molecular weight, the area under the peak, signal-to-noise ratio, peak height and peak width are reported for each named peak. The area under the peak represents the signal intensity of the immuno-detected protein.

### Qualitative analyses of CD36, Erk1/2 and Akt by Western blot and Wes

**Figure 1:**
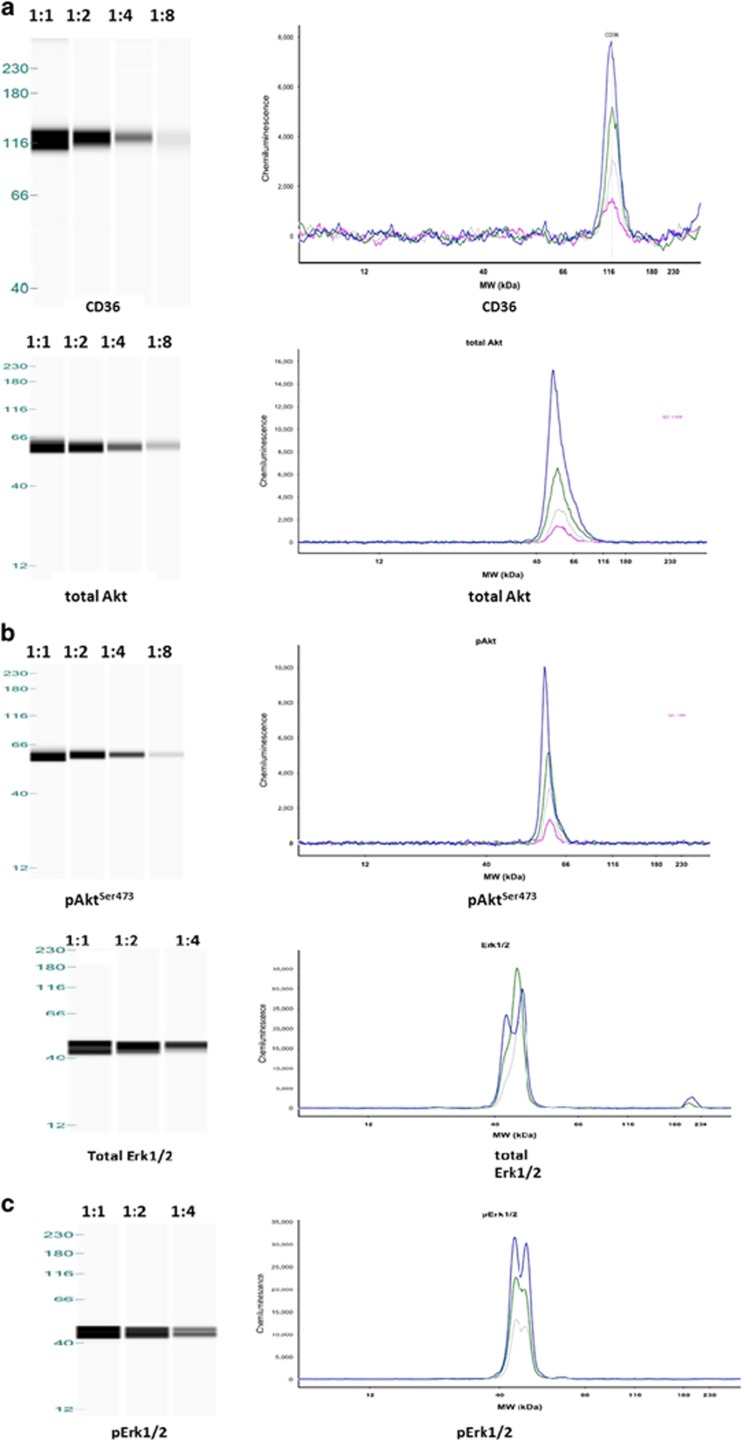
Detection of CD36 (**a**), total AKT, pAKT^S473^ (**b**) and total Erk1/2, pErk1/2 (**c**) in capillary western blot (Wes) assay, using twofold dilutions of calibrator samples. The calibrator sample was obtained from a pooled whole-tissue extract from 3 to 4 surgical waste adipose tissues. Capillary western blot results were shown as gel-like image view in left panel and electropherograms in right panel, showing decreasing intensities of bands and decreasing peak areas with serial dilutions of loading samples.

We used a whole-tissue extract of adipose tissue from surgical waste to test the protein detection by both western blot and Wes. Image results from traditional western blot provide main bands of CD36, total Erk1/2, pErk1/2 (phosphorylated Erk1/2), total Akt and pAkt^S473^(phosphorylated Akt on Ser473) as we expect. On Wes we observed distinct peaks around the expected molecular weights for these proteins (120 kDa for CD36, two peaks of 42 kDa and 44 kDa for Erk1/2, 60 kDa for Akt), with decreasing peak areas with serial dilutions of loading samples (Figure 1, right panel). Wes also could provide a gel-like image view, showing the decreasing intensities of bands with serial dilutions of loading samples (Figure 1, left panel).

### Comparison of dynamic range

**Figure 2:**
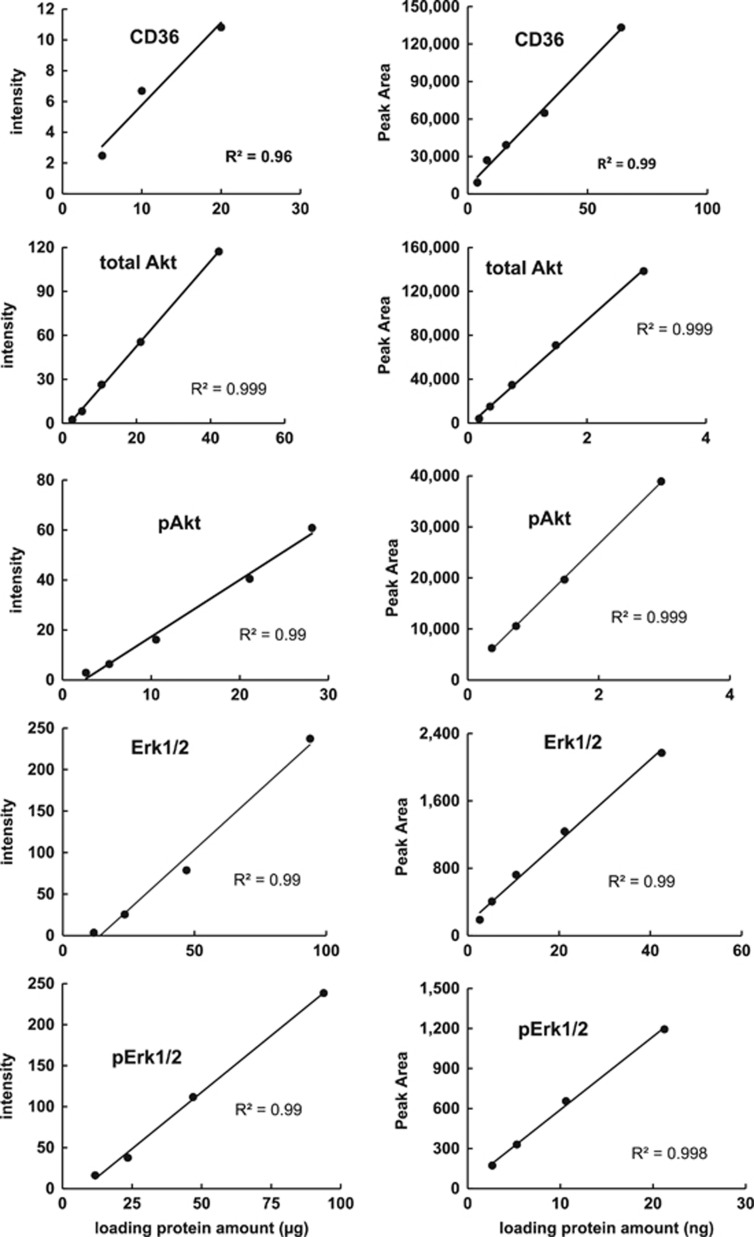
Comparison of the dynamic range of capillary western blot (Wes) assay to traditional western blot. Linear relationship between relative amounts of CD36, total Akt, pAktS473, total Erk1/2, pErk1/2 (*y* axis) and loading protein amounts (*x* axis) could be got both in western blot (left panels) and Wes (right panels) assay by using serially twofold dilution of calibrator samples. Intensities of bands in western blot and Areas under peaks in Wes (*y* axis) were detected to present for the relative amount of special proteins.

As shown in Figure 2, Both Wes and traditional western blot have linear dynamic ranges for CD36, Erk1/2 and Akt. However, Wes uses much less sample relative to traditional western blot. The linear relationship between signal intensity and total protein for the western blot was acceptable in the ranges of 5.0-20.0 μg for measuring CD36, 2.6–42.2 μg for measuring Akt and 11.7–93.9 μg for measuring Erk1/2. For the Wes the linear relationship for signal intensity was acceptable for total protein amounts of 4.0–64.0 ng for CD36, 0.2–3.0 ng for Akt and 2.7–42.5 ng for Erk1/2.

### Assay performance

We used a calibrator sample obtained from a pooled whole adipose tissue extract to test the reproducibility of Wes. Using high, medium and low dilution of a calibrator sample on every run over >4 months, we found the inter-assay CV’s for CD36 and for the ratio of pErk1/2 to total Erk1/2 were both 10% and for the ratio of pAkt^S473^ to total Akt was 15%. Using the calibrator sample 10 times on a single run we found the intra-assay CV of the ratio of pAkt^S473^ to total Akt was 14% for Wes. For reference purposes we also tested the assay performance of traditional western blotting for the ratio of pAkt^S473^ to total Akt and found it to be comparable (18%).

### Comparison of CD36 between western blot and Wes results

**Figure 3:**
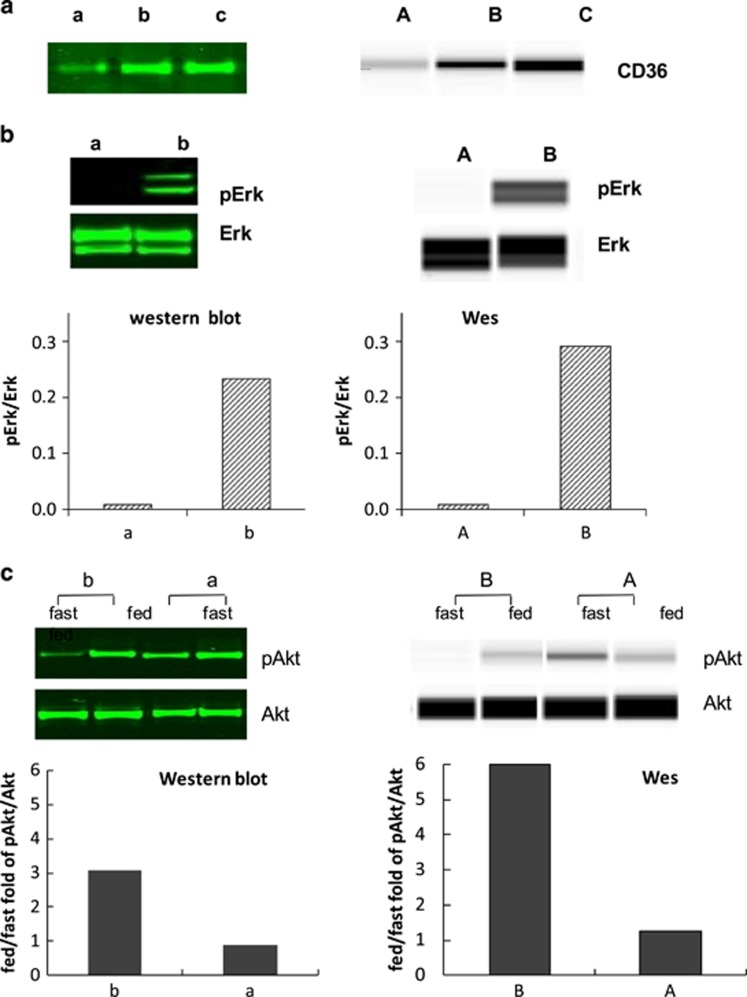
Comparison of adipose tissue samples assays in western blot (left panel) and Wes (right panel) for CD36 (**a**), Erk1/2 (**b**) and Akt (**c**). Adipose tissue samples used to detect CD36, Erk1/2 and Akt were obtained separately from 3 different studies. Either western blot or Wes showed the diverse results for relative amount of CD36, ratio of pErk1/2 to total Erk1/2 and fold increases in pAKTS473/Akt. Sample a-c and A-C represented low-high results in western blot and Wes assay accordingly.

**Figure 4:**
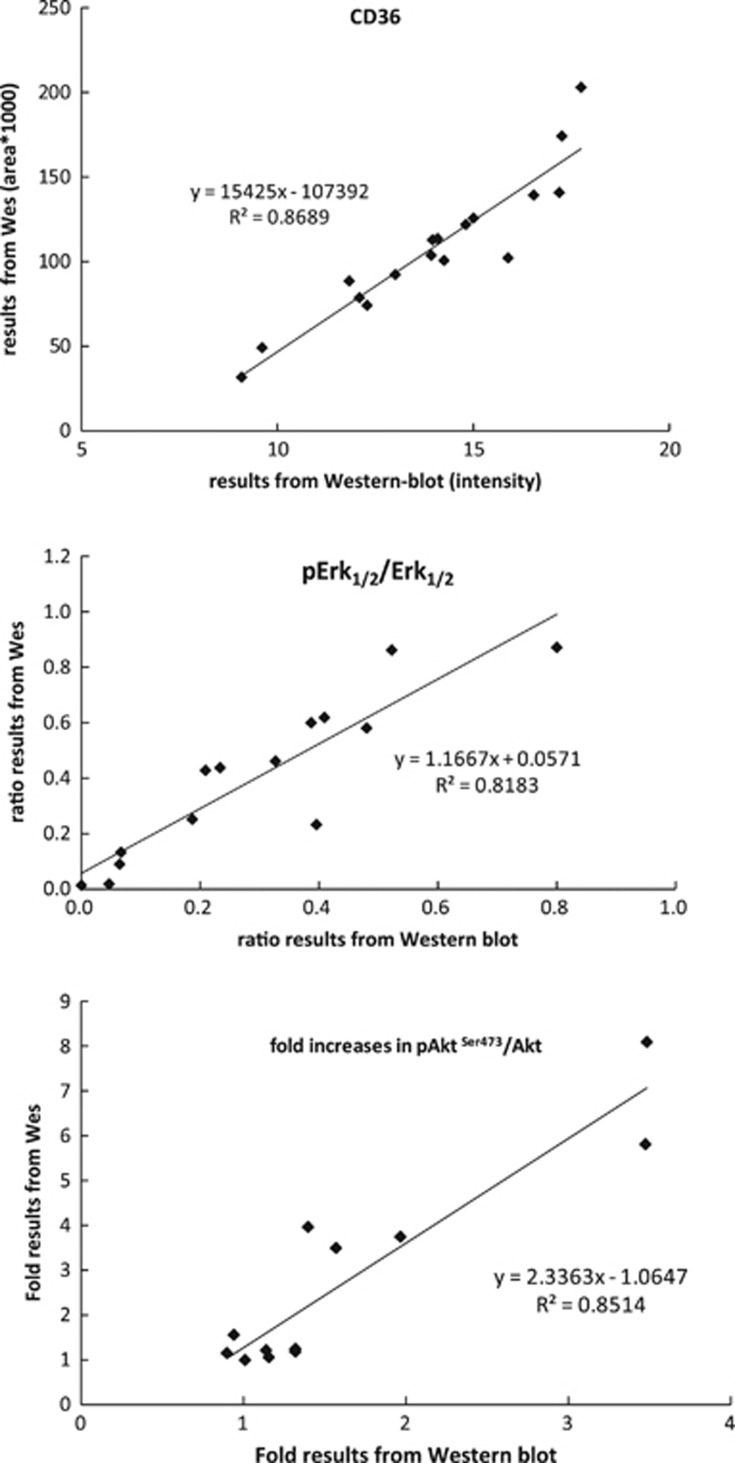
Comparison of results for relative amount of CD36, ratio of pErk1/2 to total Erk1/2 and fold increases in pAKTS473 /Akt measured using Wes and western blot. A linear regression fit was used to estimate the correlation between results got from western blot and from Wes assay. *X* axis depicted the relative amount of CD36, ratio of pErk1/2 to total Erk1/2 and fold increases in pAKTS473/Akt from western blot assays. *Y* axis depicted these results from Wes assays.

We analyzed paired 17 adipose samples (abdomen or thigh) from 7 different subjects obtained under hyperinsulinemic or hypoinsulinemic/epinephrine conditions to determine the agreement between Wes and Western in quantitating CD36 protein levels. Figure 3a depicts the examples of results from samples run on traditional western blot (left side) and Wes (right side). The results from these two methods were highly correlated (*r*=0.932, *P*<0.001, Figure 4, top panel). Using the fold results in rank ordinal numbers, the Spearman rank correlation coefficient remained significant (*r*=0.931, *P*<0.001).

### Comparison of Erk1/2 between western blot and Wes results

We analyzed paired adipose samples from 14 different subjects obtained under hyperinsulinemic or niacin exposed conditions to determine the agreement between Wes and Western in quantitating the ratio of pErk1/2 to Erk1/2. Figure 3 panel B depicts the results from 2 of these samples run on traditional western blot (left side) and Wes (right side) for pErk1/2 to Erk1/2 Wes. The results from these two methods were highly correlated (*r*=0.905, *P*<0.001, Figure 4, middle panel). Using the fold results and rank ordinal numbers, the Spearman rank correlation coefficient remained significant (*r*=0.912, *P*<0.001).

### Approaches to allow between method comparisons

**Figure 5:**
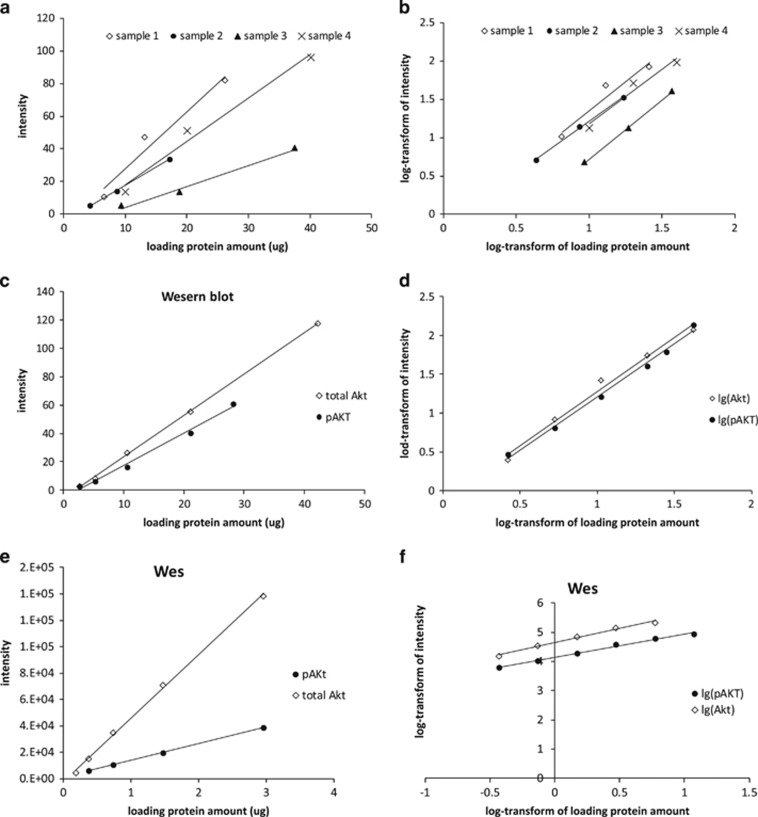
The regression lines of four adipose tissue samples for tested total Akt and QC sample for total Akt and pAkt S473 before and after log-transform in western blot and Wes analyses. (**a**) The regression lines of different adipose tissue extracts are not parallel. After log-transforming both *X* axis (loading protein amount) and *Y* axis (intensity), the regression lines of these four samples become parallel (**b**). (**c**, **e**) The regression lines of QC sample for total Akt and pAktS473 tested by western blot and Wes, (**d, f**) The regression lines of QC sample for total Akt and pAkt S473 after log-transform in western blot and Wes.

One of our goals was to determine whether changes in Akt phosphorylation are comparable using Wes and traditional western blotting. In the course of testing four different surgical waste adipose tissue samples using traditional western blot approaches we found different responses between the loading protein amount and the signal intensity for total Akt (Figure 5a). The implication of this observation is that between-sample differences in adipose tissue Akt content are not linearly related, even if identical amounts of protein are loaded. Of interest, the lines describing the relationships for these four samples were virtually parallel after log-transforming both *x* axis (loading protein amount) and *y* axis (intensity) data (Figure 5b). We also found that the relationships between protein amount and signal intensity for total Akt and pAkt^S473^ in our calibrator sample were different when plotted using unadjusted data for both traditional western blotting (Figure 5c) and the Wes (Figure 5e). These differences in the slopes of the responses would make it impossible to compare changes in the ratio of pAkt to total Akt between traditional western blotting and Wes. We found that the relationships became virtually parallel for both western blot (Figure 5d) and Wes (Figure 5f) if we log transformed the protein and signal intensity data, allowing us to compare the changes in pAkt:Akt between traditional western blotting and Wes.

### Comparison of Akt between western blot and Wes results

We analyzed paired adipose samples from 12 different subjects obtained in the fasted and fed states to determine the agreement between Wes and Western in quantitating the fold increase in pAkt/Akt. Figure 3c depicts the diverse fold increases in pAKT^S473^/Akt between the fasting and fed state for two subjects using both traditional western blot (upper) and Wes (lower) methods. The results from the two methods were highly correlated (*r*=0.923, *P*<0.001, Figure 4, bottom panel). Using the fold results a rank ordinal numbers, the Spearman rank correlation coefficient remained significant (*r*=0.797, *P*=0.002).

## Discussion

Because capillary Western approaches offer investigators the ability to measure multiple proteins and require much smaller amounts of tissue, we wished to test whether this method compares favorably to traditional western blotting for human adipose tissue proteins. We found that the ProteinSimple Wes, a more sensitive and automated approach for protein detection and characterization, provided comparable results to traditional western blotting. We also encountered some challenges that relate to nuances of signal responsiveness of the two methods. The capillary Western approach for measuring protein content (CD36), the phosphorylation state of a protein (pErk1/2:Erk1/2), and even the change in pAkt/Akt in response to meal ingestion agreed well between the two methods. We conclude that this capillary Western method of measuring proteins in adipose tissue is a valid approach that requires less tissue and is less labor intensive.

Previous studies described the application of such capillary western blot system for vaccine development,^11^ biopharmaceutical fusion-Fc protein evaluation^12^ and signal pathway modulation in cell samples.^13^ Other reported applications of this capillary Western system include specific biomarkers for diabetes and cancer research.^14^ Because we found no publications of its use for protein analyses in human adipose tissue we sought to understand whether capillary Western approaches can compete with traditional western blotting.

Traditional western blot requires many time consuming steps, including sample loading, gel electrophoresis, transfer, blocking, primary and secondary antibody incubation and detection.^15^ Because all of these steps are manual it is sometimes challenging to become proficient and maintain consistent results. However for Wes, the separation electrophoresis and immune-detection steps take place fully automated in the glass capillaries after plate loading.^16^ We found that the reproducibility of automated capillary Western approach is equal to or better than traditional western blotting, perhaps because it reduces the chance for operator error. It also requires less time for sample preparation and plate loading (~2 h) and less total run time (~3 h). Although the supply expenses are greater for capillary Western methods, the time savings and greater sensitivity (requiring less sample) are offsetting advantages.

Of the three proteins we selected for comparison, measuring the fold increase in pAkt^S473^/Akt in response to feeding was most challenging. To measure the ratio of pErk1/2 to Erk1/2, we could load the same protein amount for Erk1/2 and pErk1/2. In contrast, to be able to observe the fold increase of pAkt^S473^ we were required to load different amounts of protein for the fasting and fed samples. This was because fasted pAkt^S473^ was very low in many samples. In cases where the fasting pAkt ^S473^/Akt was at or near zero, we could not calculate the fold increase. However, loading more protein to detect pAkt^S473^ was not a viable approach because the intensity of total Akt would exceed the top of linear dynamic range, especially in Wes. To address the need to load different protein amounts for total Akt and pAkt^S473^ assays we used a calibrator sample as a quality control (QC), allowing us to calculate the relative amount of signal to the QC and thereby calculate the ratio of pAkt^S473^ to total Akt. Furthermore, by log-transforming both the loading protein amount and the signal intensity we could achieve parallel lines describing the relationships for total Akt and pAkt^S473^ in both western blot and Wes assays by testing the calibrator sample. This allowed us to compare fold increase in using the two approaches, which was not possible absent this solution.

In summary, our results indicate that capillary Western approaches using ProteinSimple Wes provides an attractive alternative for protein analyses of human adipose tissue. We found the approach to be time-saving, with high sensitivity, good quantitation and reproducibility.
